# How strong is the evidence base for carbohydrate restriction in the management of type 2 diabetes?

**DOI:** 10.1093/ajcn/nqac096

**Published:** 2022-05-10

**Authors:** Gary Frost

**Affiliations:** Department of Metabolism, Digestion and Reproduction, Faculty of Medicine, Imperial College Hammersmith Campus, London, United Kingdom

See corresponding article on page 40.

The article by Jayedi et al. (1) in the current issue of the *American Journal of Clinical Nutrition* brings new methodology and insight to a hotly debated area, namely, how to develop a dose response for carbohydrate reduction in type 2 diabetes management. Carbohydrate restriction in this setting is of course not new. Before insulin was available, the most common dietary treatment for diabetes mellitus was a high-fat, low-carbohydrate diet (2). Even after the discovery and mass production of insulin, dietary carbohydrate remained restricted in the diet of people with diabetes. It was not until the late 1970s and early 1980s, with the life expectancy of people living with diabetes increasing, that avoidance of cardiovascular complications came to the forefront of the disease management. Dietary recommendations began to appear to reduce cardiovascular risk, which included reducing fat intake and increasing carbohydrates. This coincided with a philosophical change in diabetes therapy aimed at fitting therapy around lifestyle rather than the other way around.

Glycemic control has remained a major challenge in the management of type 2 diabetes (3). Over the past 18 y, there has been a steady rise in publications relating to the efficacy of low-carbohydrate diets in type 2 diabetes (4) a number of narrative (5), systematic reviews and meta-analyses in the area (6–8), most of which suggest a positive effect on glycemic control and body weight. This accumulating evidence convinced the American Diabetes Association Standards of Care Committee in 2019 to make 2 consensus statements (9):

- Reducing overall carbohydrate intake for individuals with diabetes has demonstrated the most evidence for improving glycemia and may be applied in a variety of eating patterns that meet individual needs and preferences.
- For select adults with type 2 diabetes not meeting glycemic targets or in whom reducing antiglycemic medications is a priority, reducing overall carbohydrate intake with low- or very low-carbohydrate eating plans is a viable approach.

However, despite these recommendations, it is unclear what is meant by “low carbohydrate,” and there has been no attempt to understand whether a dose–effect relation exists between glycemic control and the extent of dietary carbohydrate restriction. These points are the major gaps that the article by Jayedi and colleagues (1) fills. The systematic review takes advantage of a new statistical methodology for meta-analysis, namely, dose–response meta-analysis of differences. This method, developed by Crippa and Orsini (10), consists of dose–response models to estimate within each study and an overall curve obtained by pooling study-specific dose–response coefficients, thus allowing a dose–response curve to be created across the systematic review [namely, Figure 1 in Jayedi et al. (1)]. This method has given the authors the ability to estimate the impact of a stepped reduction in carbohydrate intake from 55–65% of energy to 10%. [In contrast, previous studies have tended to grade carbohydrate intake more coarsely, such as moderate (≤45% to 26%) (8) (≤25% to 11%) (8), and very 68 low (≤10%) (8) intake.] The article's main results show a clear dose-related decrease in hemoglobin A1c, fasting blood glucose, triglycerides, and systolic blood pressure and body weight with an increase in HDL cholesterol after 6 mo of carbohydrate reduction. However, a U-shaped curve for total cholesterol and LDL cholesterol was noted at 6 mo, suggesting that low intake of carbohydrate and presumed increase in fat intake could have detrimental effects on cardiovascular health. Also, the relation between lower carbohydrate intake and lower body weight at 6 mo was lost at 12 mo, perhaps suggesting that long-term compliance to lower carbohydrate intake in the long term is difficult. Of interest, a recent comparison of low-carbohydrate and low-fat dietary intervention suggests adherence was similar between the diets (11).

One of the limitations of systematic reviews is the lack of mechanistic insight. Why should low-carbohydrate diets have such a profound effect across both glycemic control and lipid homeostasis? The impact on glycemic control may be as simple as the reduction in major substrate that impacts on postprandial glycemia. However, the impact on lipid fraction is more difficult to understand. This has often been ascribed to a reduction in energy intake that accompanies carbohydrate restriction. In this article, the authors conducted a subanalysis, grouping the trials “without calorie restriction” and “those with calorie-matched controls” to try to explore this issue. They state that the results were similar between these groups but qualify this statement by pointing out that most studies in the systematic review aimed to induce weight loss. However, the effect of low carbohydrate on total cholesterol and LDL cholesterol at 12 mo is lost across the dose decline in carbohydrate intake. Although unexplained, this could be due to lowered compliance given the deterioration in dose-dependent weight loss at 12 mo.

The new methodology of dose–response meta-analysis of differences used by the current article offers new insight into the impact of low-carbohydrate diets on cardiometabolic risk factors in people living with type 2 diabetes. The methodology also opens up the possibility of gaining further depth of understanding of dose response across the field of nutrition. This systematic review highlights the metabolic complexity of response to dietary intervention in type 2 diabetes as well as the need to better understand longer-term compliance and results.

## Notes

Supported with funding from the NIHR (National Institute of Health Research) Imperial Biomedical Research Centre and by the BBSRC (Biotechnology and Biological Sciences Research Council) and MRC (Medical Research Council). The views expressed are those of the author(s) and not necessarily those of the NIHR or the Department of Health and Social Care.

## References

[1] Jayedi A , Zeraattalab-Motlagh S, Jabbarzadeh B, Mousavi Y, Jibril AT, Shahinfar H et al. Dose-dependent effect of carbohydrate restriction for type 2 diabetes management: a systematic review and dose-response meta-analysis of randomized controlled trials. Am J Clin Nutr. 2022;116(1):40–56. 35537861 10.1093/ajcn/nqac066

[2] Moore MD . Food as medicine: diet, diabetes management, and the patient in twentieth century Britain. J Hist Med Allied Sci. 2018;73(2):150–67. 29514263 10.1093/jhmas/jry008 PMC6018972

[3] Ford ES , Li C, Little RR, Mokdad AH. Trends in A1C concentrations among U.S. adults with diagnosed diabetes from 1999 to 2004. Diabetes Care. 2008;31(1):102–4. 17934146 10.2337/dc07-0565

[4] Boden G , Sargrad K, Homko C, Mozzoli M, Stein TP. Effect of a low-carbohydrate diet on appetite, blood glucose levels, and insulin resistance in obese patients with type 2 diabetes. Ann Intern Med. 2005;142(6):403–11. 15767618 10.7326/0003-4819-142-6-200503150-00006

[5] Wheatley SD , Deakin TA, Arjomandkhah NC, Hollinrake PB, Reeves TE. Low carbohydrate dietary approaches for people with type 2 diabetes—a narrative review. Front Nutr. 2021;8:687658. 34336909 10.3389/fnut.2021.687658 PMC8319397

[6] McArdle PD , Greenfield SM, Rilstone SK, Narendran P, Haque MS, Gill PS. Carbohydrate restriction for glycaemic control in type 2 diabetes: a systematic review and meta-analysis. Diabet Med. 2019;36(3):335–48. 30426553 10.1111/dme.13862

[7] Turton J , Brinkworth GD, Field R, Parker H, Rooney K. An evidence-based approach to developing low-carbohydrate diets for type 2 diabetes management: a systematic review of interventions and methods. Diabetes Obes Metab. 2019;21(11):2513–25. 31347236 10.1111/dom.13837

[8] Snorgaard O , Poulsen GM, Andersen HK, Astrup A. Systematic review and meta-analysis of dietary carbohydrate restriction in patients with type 2 diabetes. BMJ Open Diabetes Res Care. 2017;5(1):e000354. 10.1136/bmjdrc-2016-000354 PMC5337734 28316796

[9] Evert AB , Dennison M, Gardner CD, Timothy Garvey W, Karen Lau KH, MacLeod J et al. Nutrition therapy for adults with diabetes or prediabetes: a consensus report. Diabetes Care. 2019;42(5):731–54. 31000505 10.2337/dci19-0014 PMC7011201

[10] Crippa A , Orsini N. Dose-response meta-analysis of differences in means. BMC Med Res Methodol. 2016;16(1):1–10. 27485429 10.1186/s12874-016-0189-0 PMC4971698

[11] Van Zuuren EJ , Fedorowicz Z, Kuijpers T, Pijl H. Effects of low-carbohydrate- compared with low-fat-diet interventions on metabolic control in people with type 2 diabetes: a systematic review including GRADE assessments. Am J Clin Nutr. 2018;108:300–31. 30007275 10.1093/ajcn/nqy096

